# Supplementary material for: The Construction and Exploration of a Comprehensive MicroRNA Centered Regulatory Network in Foxtail Millet (Setaria italica L.)
Source: Front Plant Sci. 2022 May 6;13:848474. doi: 10.3389/fpls.2022.848474 (PMC9121102; doi:10.3389/fpls.2022.848474)
Supplement: Supplementary file 5 [file Data_Sheet_5.zip › Sit-miR395j_Seita.1G205300.1_151_TPlot.pdf]

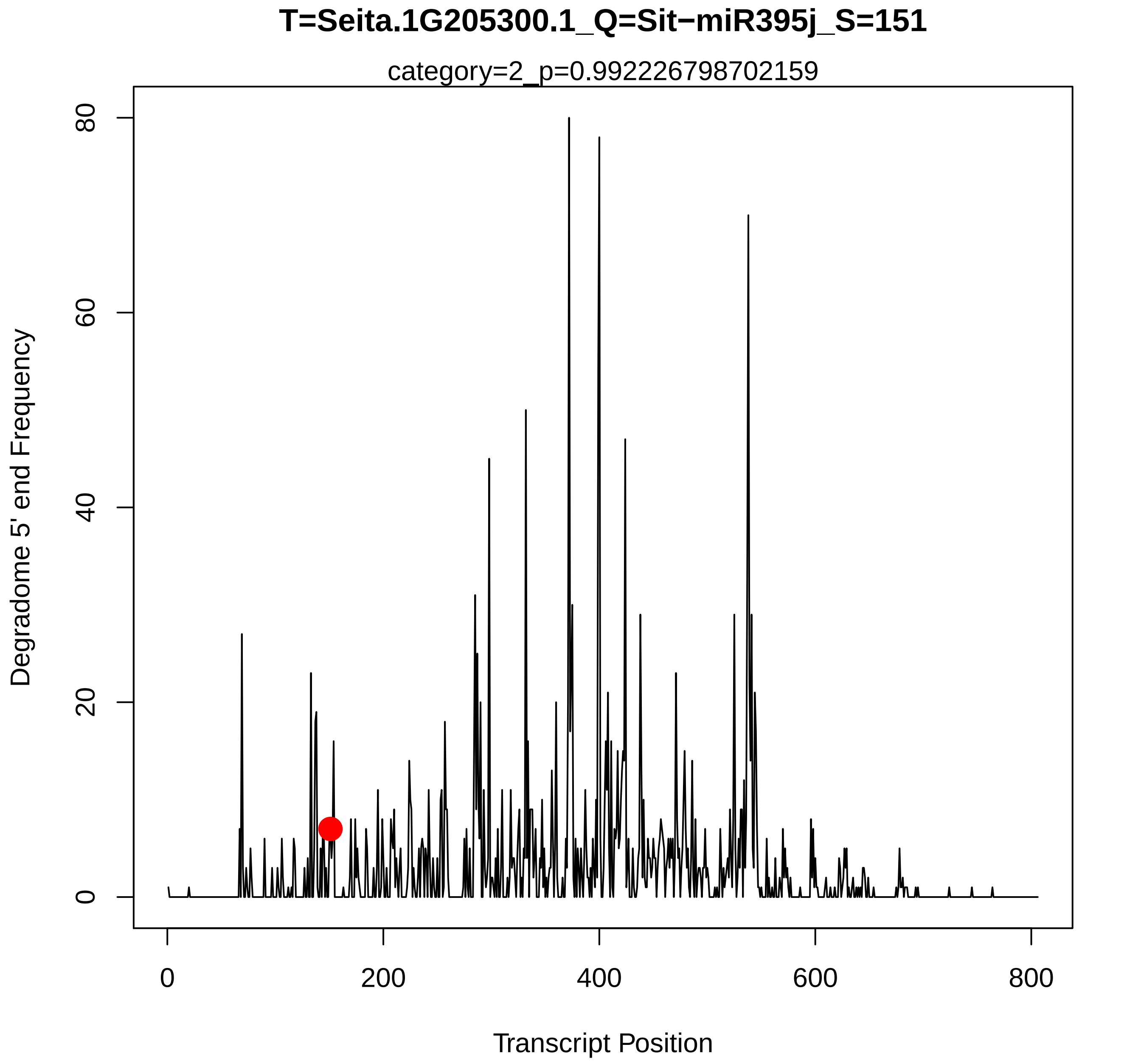
T=Seita.1G205300.1_Q=Sit−miR395j_S=151
category=2_p=0.992226798702159
Degradome 5' end Frequency
0
20
40
60
80
0
200
400
600
800
Transcript Position
